# Knowledge graph analysis of research on nurses’ psychological resilience

**DOI:** 10.1097/MD.0000000000039249

**Published:** 2024-08-09

**Authors:** Neng Liu, Jindong Yi, Fulai Yuan, Pan Su

**Affiliations:** a Teaching and Research Section of Clinical Nursing, Xiangya Hospital, Central South University, Changsha, China; b Health Management Center, Xiangya Hospital, Central South University, Changsha, China.

**Keywords:** bibliometrics, knowledge graph, nurse, psychological resilience

## Abstract

In recent years, a surge in literature on psychological nurse resilience, largely driven by the COVID-19 pandemic, has prompted the need for a comprehensive understanding of the current state and emerging trends through reliable methodologies. The purpose of this study was to analyzes the research on nurses’ psychological resilience through bibliometrics to understand the current situation, foundation, and hotspot of this research field. The Web of Science core collection database was used as the search source, and CiteSpace analysis software was employed to conduct bibliometric analysis on authors, countries, institutions, keywords, and references of nurse psychological resilience-related literature published from the establishment of the database to July 11, 2023. A total of 1060 articles were included in the final analysis. The study of nurses’ psychological resilience had been highly popular and had formed a new and important research basis in recent years. China and the United States led in the number of publications and centrality respectively, with Monash Univ and Curtin Univ as top institutions in the number of publications and centrality respectively. The authors with the highest number of publications and the most frequently cited were Rees and Connor Km respectively. The most frequently cited article was Factors Associated with Mental Health Outcomes Among Health Care Workers Exposed to Coronavirus Disease 2019 published by Lai, JB, etc. Important key keywords included mental health, resilience, stress, health, outbreak, acute respiratory syndrome, etc. The research topics in this field mainly focused on 4 aspects, including nurses’ mental health, post-traumatic stress disorder, job burnout and job satisfaction, and intervention research on psychological resilience. The results of bibliometric analysis provide direct support for future scholars to explore and determine the research direction, hot spots, and find authoritative authors and institutions. At the moment, nurses’ psychological resilience research has established a new foundation, primarily focusing on COVID-19-related topics. Given the potential prolonged coexistence of COVID-19 and other diseases, the main research focus remains innovating and validating effective psychological resilience intervention strategies for nurses’ overall well-being.

## 1. Introduction

Nurses are one of the key healthcare providers who face many pressures and challenges, especially during the COVID-19 pandemic. These pressures and challenges include high-risk work environments, long working hours, patient overcrowding, changing work demands, and concerns about their own health and that of their families.^[1]^ These factors can have a devastating impact on the physical and mental health of nurses. After analyzing cross-sectional data of 1422 health workers, Luceno-moreno et al^[2]^ found that 56.6% of health workers had symptoms of post-traumatic stress disorder (PTSD), 58.6% had symptoms of anxiety disorder, 46% had symptoms of depression, and 41.1% felt emotionally exhausted during the COVID-19 epidemic. A study by Ching et al^[3]^ found that one-third of medical workers in Asia suffer from depression, anxiety and stress during the COVID-19 pandemic, and more than two-thirds suffer from fear and burnout. In addition, epidemics/pandemics such as SARS, MERS, Ebola, and Influenza A can have a similar negative impact on nurses’ physical and mental health. A review of 44 studies on the psychological impact of epidemic/pandemic outbreaks (i.e. SARS, MERS, COVID-19, Ebola, and influenza A) on health care workers by Preti et al,^[4]^ found that 11% to 73.4% of health care workers, most of whom were nurses. They had severe symptoms of post-traumatic stress, depression, insomnia and anxiety during epidemics and pandemics.

Specifically, individual nurses exhibit distinct responses to significant public health emergencies, such as COVID-19 or other adverse conditions. Some individuals may experience pronounced stress reactions, leading to symptoms of anxiety and depression, while others remain unaffected mentally and physically. A key factor contributing to this variation is the disparity in psychological resilience among nurses. Psychological resilience refers to an individual’s ability to effectively adapt in the face of adversity, hardship, or significant stress, reflecting both personal qualities and abilities.^[5,6]^ Previous research had established a strong correlation between nurses’ psychological resilience and their susceptibility to depression,^[7,8]^ as well as their working environment conditions. Nurses with high levels of psychological resilience can adapt flexibly to their surroundings and maintain their mental and physical health. Currently, researchers were persistently exploring the specific mechanisms through which psychological resilience influences the mental and physical health of nurses. However, in order to facilitate more focused research on nurses’ psychological resilience effectively, it was crucial to possess a comprehensive understanding of the current status and trends within this academic field as it could serve as a foundation for conducting substantive studies.

In recent years, there had been a significant increase in the number of internationally published studies on nurses’ psychological resilience, leading to the emergence of numerous new and meaningful research directions. This posed challenges in objectively and comprehensively understanding the current state of this field. Relying solely on manual reading is arduous, however, bibliometrics offers an objective and reliable method for this purpose. Bibliometrics, as a branch of information science, employs quantitative analysis and research techniques.^[9,10]^ It utilizes statistics and big data to provide statistical summaries of research literature within specific fields, thereby revealing the overall development status of those fields.^[11]^ Apart from its application in this context, bibliometrics is also widely used for evaluating university research capabilities or individual strengths, understanding countries or departments with strong disciplinary research capacities, identifying potential collaboration partners, conducting institutional evaluations, and identifying talent.^[12,13]^

In the realm of nurses’ psychological resilience, previous researchers have expounded upon the current research landscape extensively. Nonetheless, there was a scarcity of comprehensive overviews that encompass the distinctive temporal period of the COVID-19 pandemic, for example, what important research hotspots, research directions, authoritative authors, and institutions in this special period have not been objectively confirmed. Furthermore, few studies have utilized bibliometric approaches to conduct qualitative and quantitative visual assessments in this field. Therefore, this study intends to adopt the method of bibliometrics to analyze the literature data related to the field of nurses’ psychological resilience, so as to find out the important research hotspots, research directions, research trends, and important research authors and institutions in this field, so as to help subsequent scholars have a comprehensive, objective and new understanding of this research field, and further promote the development of this research field.

## 2. Data and method

### 2.1. Data source

**Figure 1.**
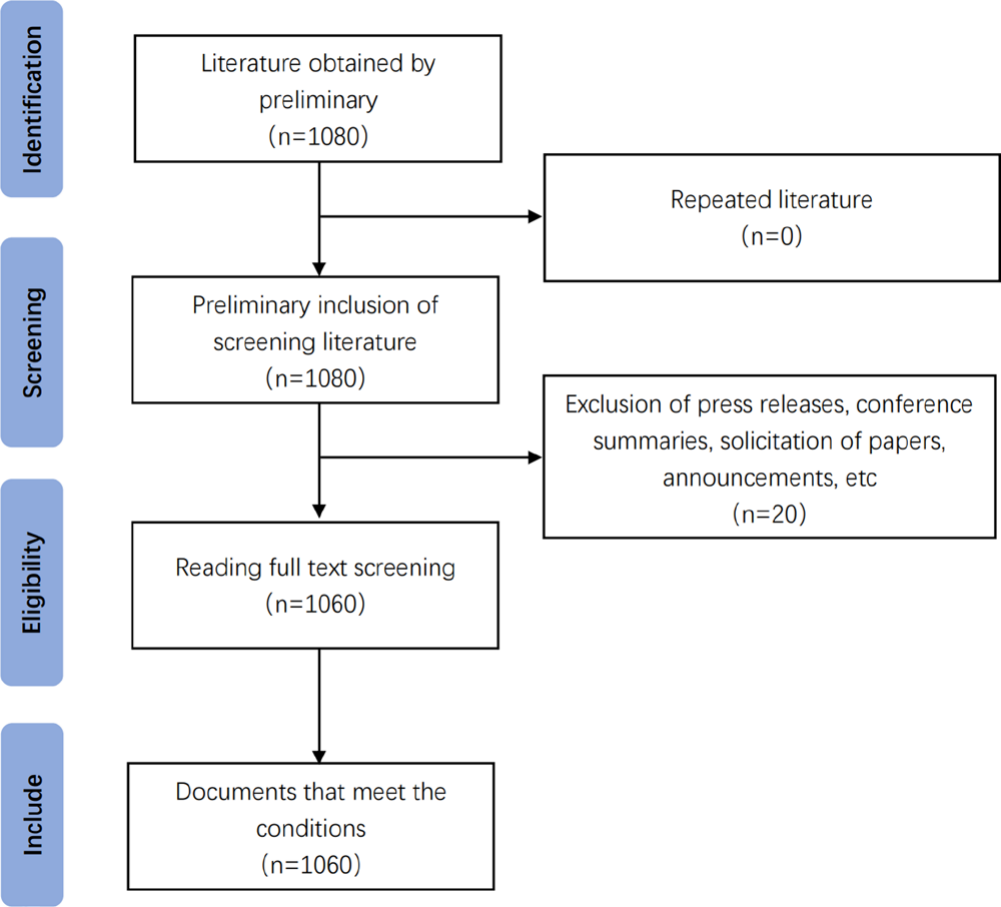
Retrieval flow chart.

Using advanced retrieval techniques, a search strategy was developed to retrieve the web of science database from its inception until July 11, 2023. The retrieval strategy for the WOS Core Collection was as follows: (TS = (Psychological Resilience) OR TS = (Resiliency, Psychological) OR TS = (Psychological Resiliency)) and (TS = Nurse), with document types limited to Articles or Reviews. A total of 1080 documents were retrieved. Titles and abstracts were read, and when necessary, full texts were reviewed. Through screening, conference papers, papers unrelated to this study, and duplicate articles were excluded. Ultimately, 1060 documents were included in this study. The specific retrieval process can be seen in Figure 1. This research involved a total of 4692 authors from 3341 institutions across 270 countries/regions.

### 2.2. Research method

CiteSpace is an information visualization software developed by Dr Chaomei Chen and his team utilizing the Java programming language. It can present the structure, law, and distribution of scientific knowledge by means of visualization, so the visualization graphs obtained by such analysis methods become scientific knowledge maps. It is used to explore the research hotspots, research frontiers, knowledge bases (key documents), main authors, and institutions of a certain research field, and to help predict the future development trend of a certain research field. By importing downloaded data into CiteSpace, the temporal scope can be set from the inception of the database until July 11, 2023, with each individual time partition spanning 1 year. Then, the publication year, country, institution, author, reference, and keywords of the literature are selected as the analysis objects, and the “Top N” strategy is adopted for the node threshold. Select the top 50 levels of most cited of occurred items from each slice. In this way, Citesapce can analyze the selected objects, so as to further analyze the annual distribution of literature, the cooperation of countries, the cooperation of institutions, the cooperation of authors, important references, and keywords. The node threshold refers to the range of data to be selected for analysis. The selection condition can be the top few percent or the data that occurs more frequently. The selection of the node threshold needs to be dynamically adjusted according to the actual analysis effect.

In the knowledge graph generated by Citespace,^[14]^ N represents the number of network nodes, E represents the number of connections, Density indicates the network density. Based on the network structure and the clarity of clustering, CiteSpace provides Modularity Q and silhouette indicators, namely module value (Q value) and average contour value (S value), which can be a basis for judging the drawing effect of the atlas. The drawing of knowledge graph needs to select different threshold values and draw many times, and select the ideal graph according to Q value and S value as the final result. A higher Modularity Q value indicates better clustering in the network, with a Modularity Q value > 0.3 indicating significant community structure. Silhouette value is used to measure network homogeneity, with a value closer to 1 reflecting higher homogeneity in the network. A Silhouette value above 0.5 suggests reasonable clustering results. Nodes represent citation rings, where different colors and sizes represent different years and citation counts respectively, indicating the historical citations received since publication. For knowledge graphs of keywords, the frequency distribution of keywords can reflect citation frequency or article count in a specific field, with areas having highest article count or most citations often representing research hotspots.^[15]^ The larger the word frequency, the larger its corresponding node in the knowledge graph. Additionally, co-occurrence between nodes can be determined by link thickness to measure their proximity relationship and color can indicate when certain word frequencies occur over time. Cluster analysis refers to grouping a set of physical or abstract objects into multiple classes composed of similar objects,^[16]^ Citespace can classify the data in the field of nurses’ psychological resilience by using its inherent cluster analysis algorithm, so as to facilitate the author to find out the main research hotspots and research directions. The larger the word frequency, the larger its corresponding node in the knowledge graph. Cluster analysis refers to grouping a set of physical or abstract objects into multiple classes composed of similar objects.

## 3. Result

### 3.1. Annual number of published articles

**Figure 2.**
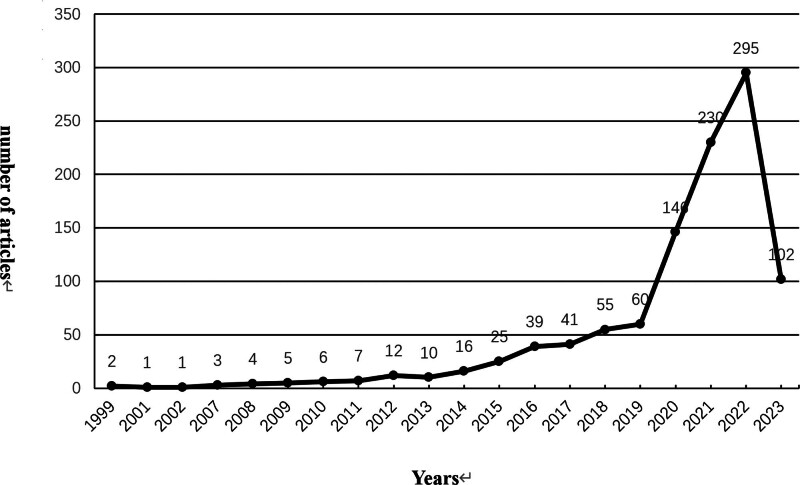
Analysis of annual publications.

There has been a substantial and rapid growth in the number of publications pertaining to nurses’ psychological resilience. This growth became particularly notable in 2019, reaching its zenith in 2022 with 295 publications. Unfortunately, comprehensive data for 2023 were not yet available due to search time constraints, but the number of literature publications was lower than this time last year. Remarkably, since 2019, there has been a significant surge in literature focusing on nurses’ psychological resilience, which could potentially be attributed to the emergence of the COVID-19 pandemic. The results of an annual number of published articles can be seen in Figure 2.

### 3.2. National distribution

**Figure 3.**
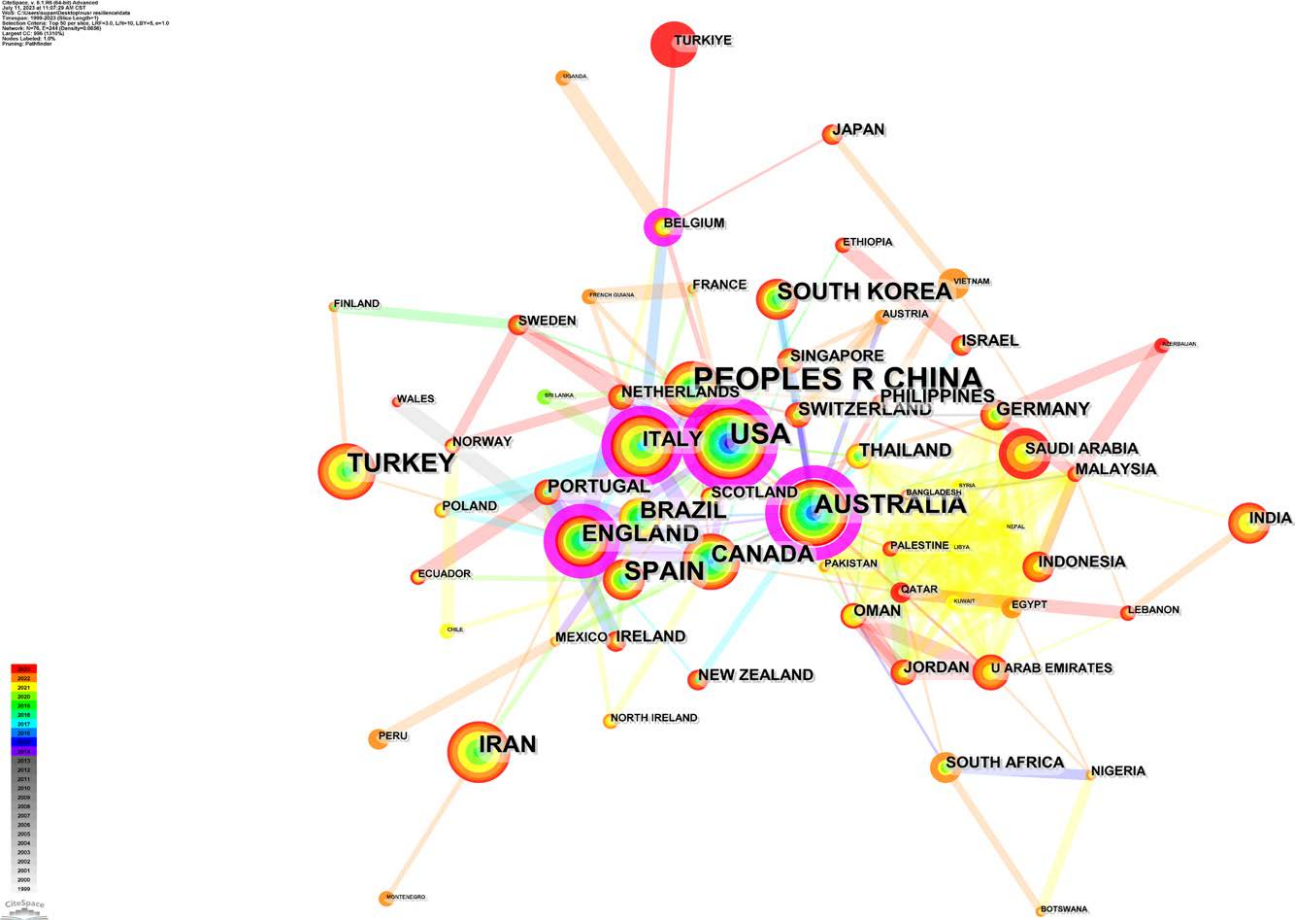
The knowledge map of collaborating countries.

In terms of publication volume, China, the United States, and Australia emerge as the top 3 countries, their publications were 292, 229 and 140, respectively. This is also shown in the knowledge graph, for example, the width of their rings in the knowledge graph is larger than that of other countries, and the colors are mostly red, yellow, and green, indicating that there are newer and more literatures published in the field of nurses’ psychological resilience in these countries in recent years. The reason may be related to the earlier and more severe impact of the COVID-19 pandemic in these countries, which had promoted the development of research in this field in these countries to a certain extent. However, when considering centrality rankings, Australia, the United States, and England take the lead, their centrality values were 0.45, 0.26, and 0.24, respectively. The results of the top 10 countries in terms of publication volume and centrality can be seen in Table 1. Figure 3 illustrates a knowledge graph depicting international collaboration networks among these nations. It is not difficult to see from the knowledge map that the connections among countries are relatively close, most of the lines are thicker, and the color of the connections is mostly yellow, orange and red, indicating that the cooperation among them is relatively close, and most of them are concentrated in 2019 and after.

**Table 1:** Countries distribution.

Country	Count	Country	Centrality
Peoples R China	292	Australia	0.45
USA	229	USA	0.26
Australia	140	England	0.24
Spain	108	Belgium	0.11
Turkey	92	Italy	0.1
England	89	Malaysia	0.09
Iran	72	Palestine	0.09
South Korea	71	Peoples R China	0.08
Brazil	59	Spain	0.08
Canada	57	Thailand	0.08

### 3.3. Institution distribution

**Figure 4.**
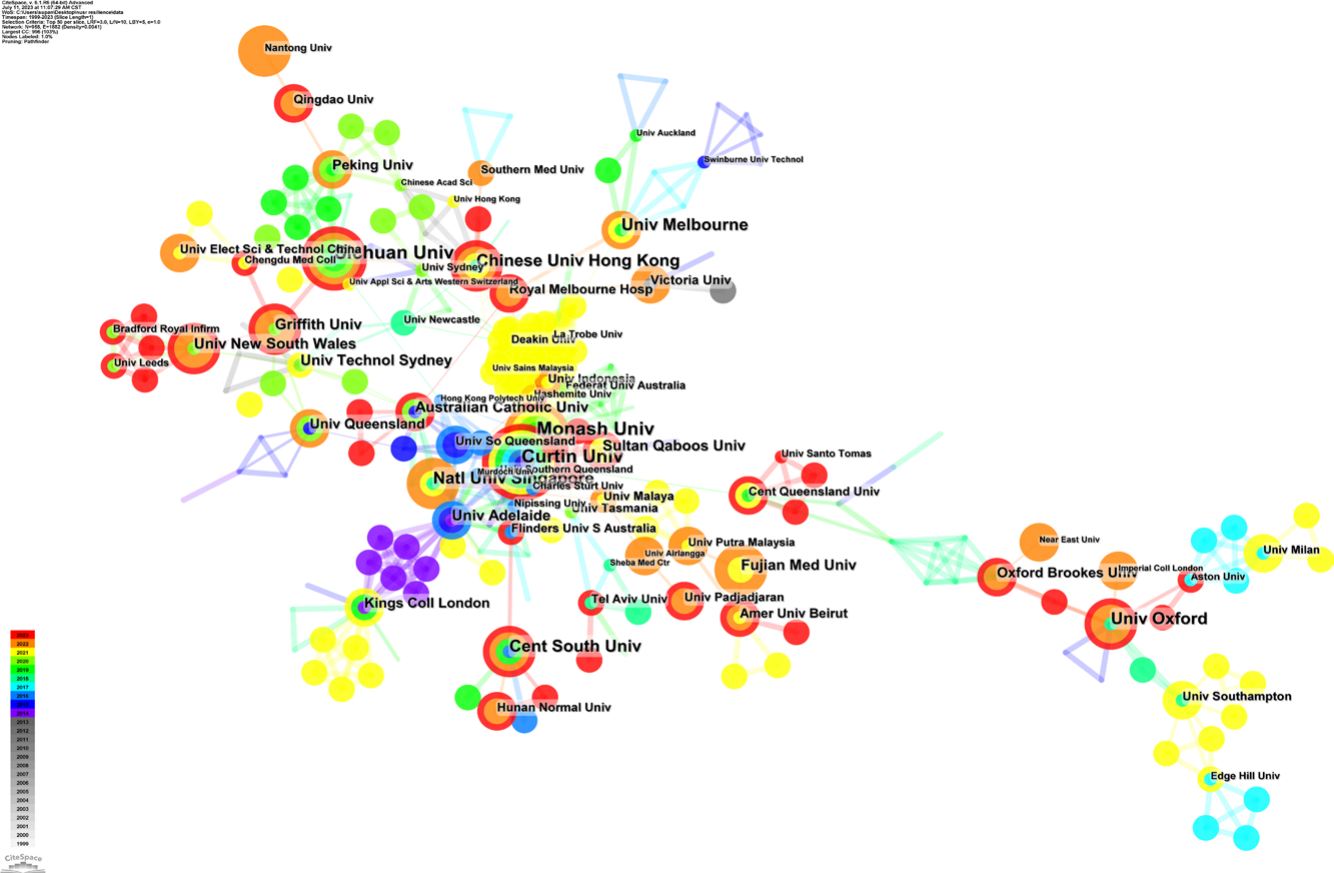
The knowledge map of collaborating institutions.

The leading 3 institutions in terms of publication volume are Monash University, Curtin University, and Shandong University, they all published 18 articles. The top 3 institutions in terms of centrality rankings are Curtin University, Monash University, and Central Queensland University, their centrality values were 0.04, 0.03, and 0.03, respectively. The results of the top 10 institutions in terms of publication volume and centrality can be seen in Table 2. Figure 4 depicts a knowledge graph of institutional collaboration networks. From this graph, it is evident that collaborations among different institutions are close-knit, similar to inter-country collaborations. Furthermore, the intensity of collaboration among institutions had been particularly noticeable in recent years.

**Table 2:** Institutions distribution.

Institution	Count	Institution	Centrality
Monash Univ	18	Curtin Univ	0.04
Curtin Univ	18	Monash Univ	0.03
Shandong Univ	18	Cent Queensland Univ	0.03
Sichuan Univ	17	Univ Oxford	0.02
Univ Seville	15	Oxford Brookes Univ	0.02
Chinese Univ Hong Kong	14	Univ Otago	0.02
Natl Univ Singapore	14	Sichuan Univ	0.01
Univ Valencia	14	Chinese Univ Hong Kong	0.01
Minist Hlth	14	Natl Univ Singapore	0.01
Univ Sao Paulo	14	Univ Melbourne	0.01

### 3.4. Author distribution

**Figure 5.**
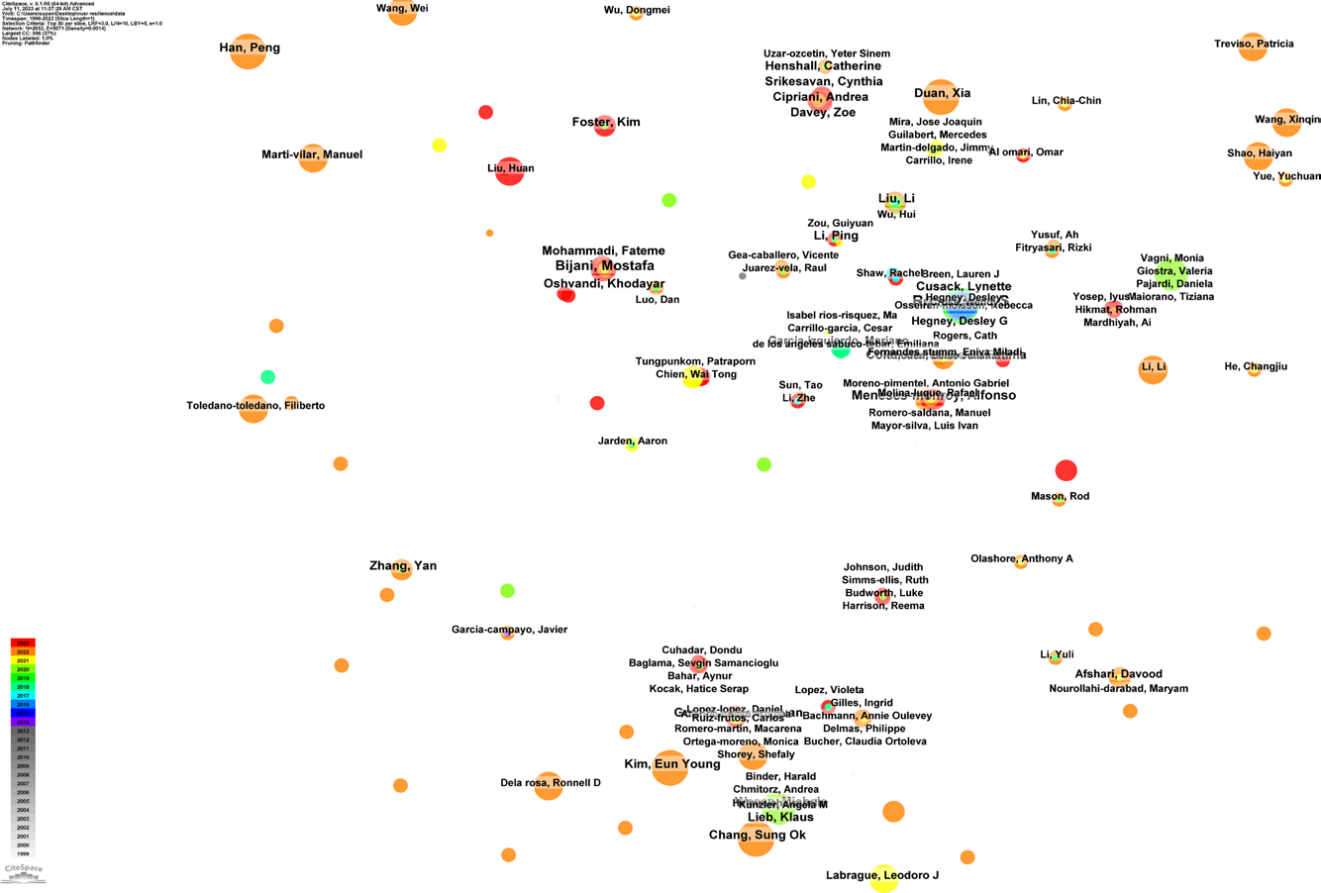
The knowledge map of collaborating authors.

The top 3 authors in terms of publication frequency are Rees, Clare S., Bijani, Mostafa, and Meneses-monroy, Alfonso, the number of their publication was 10, 8 and 7 respectively. As for citation frequency, the top 3 authors are Connor Km, Lai Jb, and World Health Organization, the cited frequencies were 327, 315 and 190 times, respectively. The results of the top 10 authors in terms of publication volume and citation frequency can be seen in Table 3. Figure 5 presents a knowledge graph visualizing author collaboration networks. The nodes in the graph appear relatively dispersed, suggesting limited collaboration among the authors, all of whom possess a centrality score of zero.

**Table 3:** Authors distribution.

Author	Count	Author	Centrality
CONNOR KM	327	AMERICAN PSYCHIATRIC ASSOCIATION	0.3
LAI JB	190	ANTONOVSKY A	0.24
UNKNOWN	183	BANDURA A	0.21
WORLD HEALTH ORGANIZATION	173	LAZARUS R. S	0.18
LABRAGUE LJ	169	ANTONOVSKY A	0.18
MEALER M	139	TEDESCHI RG	0.17
CAMPBELL-SILLS L	128	MASLACH C	0.14
PAPPA S	117	AHOLA K	0.1
SMITH BW	114	ARNOLD D	0.09
MASLACH C	113	LUTHAR SS	0.08

### 3.5. Highly cited literature

The 3 most frequently cited articles are as follows: “Factors Associated With Mental Health Outcomes Among Health Care Workers Exposed to Coronavirus Disease 2019” by Lai et al, published in the JAMA NETWORK OPEN journal; “Prevalence of depression, anxiety, and insomnia among healthcare workers during the COVID-19 pandemic: A systematic review and meta-analysis” by Pappa et al, published in the BRAIN BEHAVIOR AND IMMUNITY journal; and “Managing mental health challenges faced by healthcare workers during the COVID-19 pandemic” by Greenberg et al, published in the BMJ-BRITISH MEDICAL JOURNAL. The top 10 highly cited references can be seen in Table 4.

**Table 4:** Cited reference distribution.

Cited reference	Count
Lai JB, 2020, JAMA NETW OPEN, V3, P0, DOI 10.1001/jamanetworkopen.2020.3976	190
Pappa S, 2020, BRAIN BEHAV IMMUN, V88, P901, DOI 10.1016/j.bbi.2020.05.026	61
Greenberg N, 2020, BMJ-BRIT MED J, V368, P0, DOI 10.1136/bmj.m1211	61
Labrague LJ, 2020, J NURS MANAGE, V28, P1653, DOI 10.1111/jonm.13121	57
Pappa S, 2020, BRAIN BEHAV IMMUN, V88, P901, DOI 10.1016/j.bbi.2020.05.026, 10.1016/j.bbi.2020.11.023	54
Chen QN, 2020, LANCET PSYCHIAT, V7, PE15, DOI 10.1016/S2215-0366(20)30078-X	49
Yu F, 2019, INT J NURS STUD, V93, P129, DOI 10.1016/j.ijnurstu.2019.02.014	48
Sun NN, 2020, AM J INFECT CONTROL, V48, P592, DOI 10.1016/j.ajic.2020.03.018	47
Kang LJ, 2020, BRAIN BEHAV IMMUN, V87, P11, DOI 10.1016/j.bbi.2020.03.028	45
Guo YF, 2018, J CLIN NURS, V27, P441, DOI 10.1111/jocn.13952	44

### 3.6. Keyword distribution

**Figure 6.**
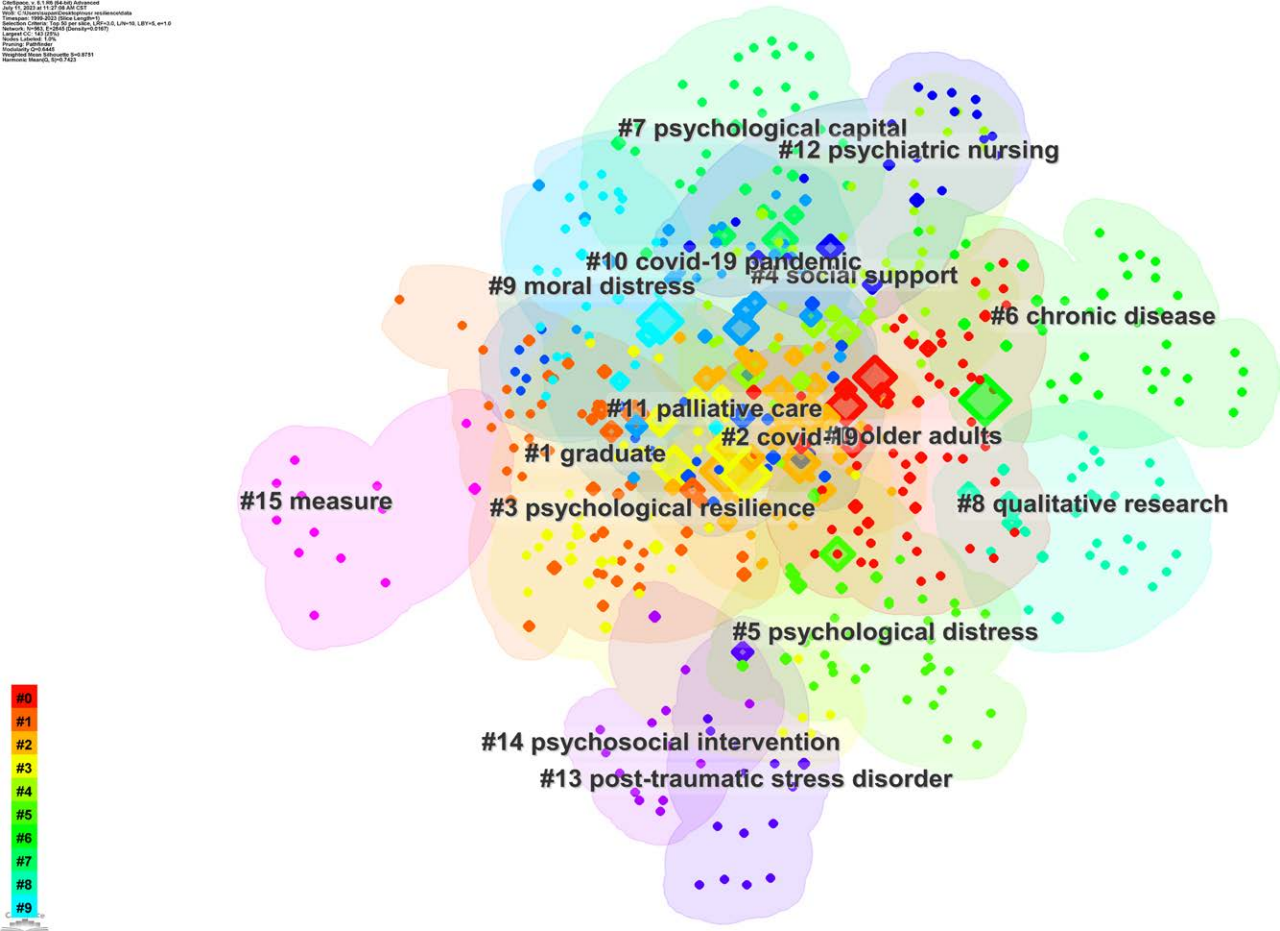
The knowledge map of keyword clustering.

The top 10 keywords in terms of frequency are mental health, resilience, stress, nurse, psychological resilience, burnout, health, depression, scale and impact. In terms of centrality analysis, the top 10 keywords include health, resilience, stress, PTSD, nurse, burnout, impact, quality of life, children and experience. The specific results can be observed in Table 5. Figure 6 illustrates the network knowledge graph depicting keyword clustering with a total of 16 clusters obtained. The specific cluster labels can be seen in Figure 6. The Modularity Q value of the knowledge graph is >0.3 and the Silhouette value is close to 1. This indicates that the proposed network knowledge graph structure of keyword clustering is significant and clear and suitable for analysis. The overlapping clusters in the graph indicate that research topics within this field exhibit relative concentration and similarity in content.

**Table 5:** Keywords distribution.

Keyword	Count	Keyword	Centrality
mental health	320	health	0.26
resilience	313	resilience	0.23
stress	301	stress	0.11
nurse	252	posttraumatic stress disorder	0.11
psychological resilience	216	nurse	0.1
burnout	213	burnout	0.1
health	210	impact	0.1
depression	183	quality of life	0.1
scale	181	children	0.1
impact	169	experience	0.09

**Figure 7.**
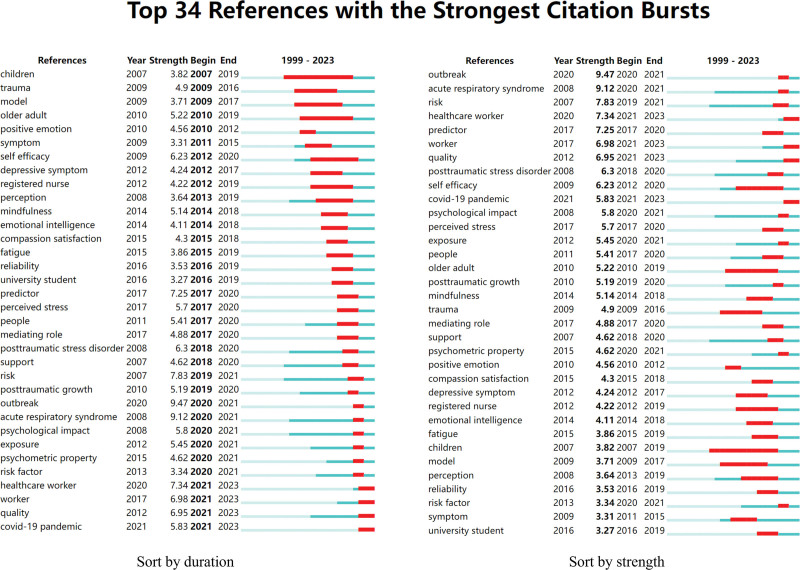
Keywords with the strongest citation bursts.

To further explore the research frontier in this field, this study conducted a keyword burst analysis and identified 34 burst keywords. The distribution of these keywords can be seen in Figure 7. The top 10 burst keywords are outbreak, acute respiratory syndrome, risk, healthcare worker, predictor, worker, quality, PTSD, self-efficacy and COVID-19 pandemic. Keywords with sustained bursts until 2023 include healthcare worker, worker, quality and COVID-19 pandemic.

## 4. Discussion

### 4.1. Analysis of research trends and international collaboration

Since the inception of the COVID-19 pandemic in 2019, there had been a considerable escalation in research concerning nurses’ psychological resilience. The reason may be that during the COVID-19 pandemic, a greater awareness of the critical role of nurses in healthcare and the important responsibilities they have led researchers to pay more attention to nurses’ work environment, mental health and ability to cope with stress. Secondly, the epidemic had put nurses under unprecedented challenges and pressure, which had further drawn attention to their mental health problems. China, the United States, Australia, and other nations had made substantial contributions to the field of nurses’ psychological resilience research. Although China had published the highest number of articles, its centrality ranking was relatively lower compared to countries like Australia, the USA, and England, which possess institutions with high publication output or centrality. This suggests that China’s international influence in this research area was not as robust as those countries with higher centrality rankings. Overall, collaborations among different nations had predominantly occurred after 2019 and indicated close cooperation among nations and institutions. Through international collaboration, academics can explore key elements of psychological resilience and effective coping strategies across geographical and cultural boundaries, thereby facilitating the sharing of knowledge, resources and best practices so that academics around the world can learn from each other’s experiences to benefit the well-being of nurses around the world and enhance their ability to cope with stress and challenges in the workplace. Ultimately improving the quality of patient care and the level of medical services.

### 4.2. Analysis of important research forces and research directions

In the knowledge graph, all authors have a centrality score of 0, indicating the absence of influential authors in the field of nurses’ psychological resilience research. Moreover, the collaboration network among authors exhibits significant dispersion and weakness, implying a lack of robust collaborative efforts. However, there are still some closely-knit and prolific academic groups identified in the knowledge graph, such as the group composed of Rees, Clare S and Cross, Wendy, Cusack, Lynette and Hegney, Desley G who are all from Australia. Their research primarily focused on investigating the impact of psychological resilience on nurses’ mental and physical health as well as their quality of professional life.^[17–20]^ One study conducted by this group revealed that psychological resilience can moderate the relationship between nurses’ neuroticism, mindfulness, self-efficacy, coping strategies, and psychological adjustment.^[21]^ Another group consists of authors from the UK including Srikesavan, Cynthia; Davey, Zoe; Henshall, Catherine; and Cipriani, Andrea. Their research direction mainly focused on analyzing factors influencing nurses’ mental and physical health during the COVID-19 pandemic from a perspective of psychological resilience along with evaluating specific intervention measures.^[22,23]^ One study conducted by this group demonstrated that implementing nurse resilience-enhancing online training programs with similar design features in busy healthcare environments was acceptable to participants and considered useful. Nurses showed enthusiasm for implementing such programs to optimize their resilience levels as well as improve their mental health status communication skills and work environment.^[24]^ In addition, a team of Chinese researchers led by Shao Haiyan, Han Peng, Jiang Jinxia, Duan Xia, and Liu Yue had dedicated their research to exploring the factors that influence the psychological resilience of critical care or emergency nurses during the COVID-19 pandemic. Their studies had demonstrated that adequate pre-outbreak preparation, well-planned arrangements, a supportive working environment built on trustworthiness, encouragement and enhancement of individual and collective coping strategies for nurses in facing adversity, timely recognition and rewards, and empowerment of nurses through counseling and training can effectively enhance their psychological resilience.^[25–27]^ Other prolific academic groups included an Iranian team consisting of Bijani, Mostafa, Mohammadi, Fateme, Oshvandi, Khodayar,^[28,29]^ as well as a Chinese team composed of Wang Xinqin Liao Yuan Ye Zengjie Mei Xiaoxiao.^[30]^ These highly connected and productive academic groups play a key role in the field of nurses’ psychological resilience research. Their research is critical to understanding the role of psychological resilience in nurses’ physical and mental health and quality of professional life. By exploring the relationship between psychological resilience and factors such as nurses’ neuroticism, mindfulness, and self-efficacy, these groups provide important clues for the development of effective interventions. In addition, they also analyzed the impact of COVID-19 on nurses’ psychological resilience and proposed corresponding coping strategies. These studies provide theoretical and practical guidance for improving nurses’ mental health. These groups will continue to be important research forces in the field in the future, and other scholars may consider collaborating with them to advance the field of nurse resilience.

### 4.3. Analysis of research basis

Prior to the COVID-19 pandemic, there was relatively limited research on nurses’ psychological resilience, as evidenced by the lack of core articles in terms of citation frequency and publication volume. However, since the outbreak of COVID-19, a substantial number of studies focusing on nurses’ psychological resilience had emerged. The knowledge graph analysis reveals that these emerging studies had formed a crucial research foundation in the field. The new knowledge base primarily centers around investigations, systematic evaluations, and interventions related to nurses’ psychological resilience. Within this newly established knowledge base, several noteworthy publications deserve mention. For instance, one highly cited article published by Lai et al^[31]^ in JAMA NETW OPEN conducted a cross-sectional study involving 1257 healthcare professionals from 34 hospitals with fever clinics or wards. The study discovered that a significant proportion of healthcare workers reported symptoms such as depression, anxiety, insomnia, and distress—particularly among female nurses and those directly involved in diagnosing or treating suspected or confirmed COVID-19 patients. Subsequent studies consistently support these findings.^[32–34]^ Next, a systematic review and meta-analysis study conducted by Pappa et al^[35]^ was published in the journal BRAIN BEHAV IMMUN. Their research indicated that a significant number of healthcare workers experienced emotional and sleep disorders during this pandemic. They emphasized the necessity to develop strategies for mitigating mental health risks and adjusting intervention measures during major outbreaks. The third most cited article was conducted by Greenberg et al, highlighting the heightened risk of moral injury and psychological health issues faced by healthcare workers in dealing with the challenges posed by the COVID-19 pandemic. The study proposed measures that healthcare managers should implement to safeguard the mental health of healthcare professionals who were confronted with morally challenging decisions.^[36]^ Moreover, numerous scholars in this field had also conducted research from the perspective of identifying protective factors for nurses’ psychological resilience. For instance, Labrague and De Los Santos^[37]^ systematic review demonstrated that coping behaviors, resilience, and social support can effectively maintain the psychological health of healthcare workers during the COVID-19 pandemic. Moreover, numerous scholars in this field had also conducted research from the perspective of identifying protective factors for nurses’ psychological resilience. For instance, Bohlken J et al’s^[38]^ systematic review demonstrates that coping behaviors, resilience, and social support can effectively maintain the psychological health of healthcare workers during the COVID-19 pandemic. Studies like these collectively contribute to establishing a solid research foundation on nurses’ psychological resilience which serves as an important groundwork for future studies in this field.

### 4.4. Important research hotspots and frontiers

The keywords that exhibit high frequency and centrality rankings in the field of nurses’ psychological resilience encompass nurse, psychological resilience, mental health, depression, breakdown, and quality of life. These keywords epitomize the current research focal points within the realm of nurses’ psychological resilience. Conversely, burst keywords such as COVID-19 pandemic, PTSD, acute respiratory syndrome, risk, predictor, and quality represent cutting-edge research areas.

Through keyword clustering analysis, we had derived 16 clusters exhibiting substantial thematic similarity. By amalgamating the aforementioned focal points and burst keywords with specific literature readings, we had discerned that these clusters predominantly concentrate on 4 aspects: nurses’ mental health, PTSD, job burnout and job satisfaction, and intervention research on psychological resilience.

#### 4.4.1. The association between psychological resilience and mental health

One of the research hotspots in the field of nurses’ psychological resilience is the relationship between psychological resilience and nurses’ physical and mental health. Research had shown that nurses with higher psychological resilience cope better with challenges and stress and recover more easily from negative emotions, making them better able to cope with the shock of an epidemic or pandemic. Labrague and De Los Santos^[37]^ study revealed that nurses exhibiting strong psychological resilience and receiving higher levels of organizational and social support were more likely to report reduced anxiety levels related to COVID-19. Coping behaviors, psychological resilience, and social support had proven effective in preserving the mental and spiritual health of healthcare workers during the COVID-19 pandemic.^[39]^ Cai et al’s^[40]^ survey involving 1521 healthcare workers found that adequate training, professional experience, adaptability, and social support were essential for first-time participants in public health emergencies among healthcare workers. A study conducted by Zou et al^[41]^ among 366 female nurses in China revealed that 85.5% of the nurses experienced psychological distress. The findings demonstrated a negative correlation between psychological resilience and both psychological distress and burnout. Furthermore, it was revealed that psychological resilience partially mediated the relationship between emotional exhaustion, depersonalization, and dimensions of psychological distress. Similar studies investigating the association between psychological resilience and nurse mental health consistently demonstrated that nurses with higher levels of psychological resilience were better able to face challenges and stressors, recover from negative emotions more easily, thus enabling them to cope better with the impact of epidemics/pandemic waves.^[42–44]^ These findings enhance our understanding of nurses’ levels of psychological resilience and provide valuable support as well as resources to assist them in effectively managing stress for improved physical and mental health.

#### 4.4.2. The association between psychological resilience and PTSD in nurses

Psychological resilience and PTSD were significant concerns for nurses during disasters or public health events, as they often face considerable psychological stress and challenges. The investigation of these issues can offer valuable insights into the mental states and adaptive mechanisms of nurses during crises, thereby contributing to the promotion of nurses’ mental health, enhancement of teamwork and coordination, improvement in nursing education and training, as well as driving advancements in the healthcare system. Vagni et al conducted a survey of Italian medical personnel involved in the initial phase of the pandemic and found that direct contact with COVID-19 patients, female gender, emergency situations, and inadequate personal protective equipment were risk factors for acute stress reactions. However, psychological resilience and coping strategies played a protective role in mitigating the impact of stress on secondary trauma.^[45]^ Mealer et al’s survey of 744 intensive care unit (ICU) nurses revealed that 22% were highly adaptable. Notably, these nurses with high levels of psychological resilience exhibited significantly lower prevalence rates of PTSD symptoms, anxiety or depression symptoms, and burnout syndrome.^[46]^ In addition to conducting research on psychological resilience and PTSD among nurses during the COVID-19 pandemic, Carmassi et al comprehensively reviewed studies examining PTSD in healthcare workers during 3 major coronavirus outbreaks over the past 2 decades. The findings revealed that various factors such as exposure level, job role, years of work experience, social and job support, work organization, isolation, age, gender, marital status, and coping style were strongly associated with both PTSD and resilience.^[47]^ Furthermore, intervention research focusing on enhancing nurses’ resilience to reduce the risk of developing PTSD had emerged as an important area of investigation. For instance, Mealer et al^[48]^ conducted a 12-week randomized controlled study, which demonstrated that resilience training for nurses, including educational workshops, written exposure sessions, event-triggered counseling sessions, mindfulness-based stress reduction exercises, and prescribed aerobic exercise programs, had positive effects in reducing PTSD symptoms. However, research consistently indicated that higher levels of psychological resilience among nurses were linked to lower levels of PTSD. Therefore, future studies could explore the stress coping mechanisms employed by highly resilient nurses to identify and develop corresponding intervention strategies.

#### 4.4.3. The association between psychological and job burnout and job satisfaction

Nurses had consistently been at high risk for job burnout. The COVID-19 pandemic had led to a variety of mental health challenges for this group, further impacting their job satisfaction. These health issues posed detrimental effects on the long-term development of healthcare services as well as the physical and mental health of nurses. Consequently, scholars had conducted pertinent studies to furnish evidence that fosters nurses’ psychological resilience while mitigating their job burnout and enhancing satisfaction levels. Dubale et al found that during the pandemic, while all healthcare providers experienced high levels of burnout, nurses reported the highest levels. Nurse burnout was associated with their work environment, interpersonal and professional conflicts, emotional distress, and lack of social support. Therefore, it was necessary to implement programs that raise awareness about burnout symptoms and provide stress management training to enhance resilience among nurses.^[49]^ Larrabee et al^[50]^ conducted a survey involving 464 registered nurses employed in 5 acute care hospitals in West Virginia, revealing that psychological resilience predicted psychological empowerment, situational stress, and job satisfaction among nurses. Shahrbabaki et al^[51,52]^ demonstrated a positive relationship between psychological resilience and work engagement during the pandemic period. Enhancing frontline nurses’ level of psychological resilience can contribute to improving their job satisfaction and ultimately, nursing quality overall. It was clear that nurses’ psychological resilience was closely linked with both job burnout and job satisfaction outcomes. In future scenarios involving epidemic diseases like COVID-19, hospital administrators and nursing managers should consider the role of nurses’ psychological resilience in mitigating potential instances of job burnout.

#### 4.4.4. Intervention study on psychological resilience of nurses

Intervention research on nurses’ psychological resilience has become an important research direction in the field of nursing, especially during the COVID-19 pandemic, when nurses are facing serious mental health problems. These problems include anxiety, stress, depression, PTSD and insomnia, etc. Therefore, active psychological interventions are needed to enhance the psychological resilience of nurses.^[53]^ Studies have shown that interventions such as cultivating happiness, improving mindfulness-based stress reduction, accepting and expressing emotional skills can effectively improve the level of psychological resilience of nurses, reduce job burnout and improve the quality of nursing practice. Koprowski et al^[54]^ conducted an intervention study involving 419 registered nurses, which demonstrated that utilizing exercise manuals as a means of fostering joy can effectively bolster the psychological resilience of nurses. This approach not only mitigated nurse burnout but also enhanced their ability to provide exceptional quality nursing care. Furthermore, Wang et al^[55]^ successfully enhanced the psychological resilience of nursing interns in China by offering online modified mindfulness-based stress reduction training. Psychological resilience interventions had been found to be effective in enhancing nurses’ psychological resilience by helping them explore the underlying thoughts behind their emotions, thereby mitigating the negative impact of these emotions on their physical and mental health.^[56]^ Additionally, various strategies such as personal stress management, meditation, yoga, acupuncture, gratitude diary keeping, participation in chorus activities, workload reduction measures, job crafting initiatives and peer networks can contribute to improving nurses’ resilience levels and promoting their overall health.^[57]^ However, it is important to acknowledge that some of these studies may have certain limitations in terms of study design which could potentially influence the assessment of intervention effectiveness.^[57]^ Given the potential long-term coexistence of COVID-19 and other emerging diseases, there is a need for innovative and validated psychological resilience interventions specifically tailored for nurses to enhance their level of psychological resilience.^[58]^

## 5. Conclusion

In recent years, the psychological resilience of nurses had been widely concerned by the society. At present, a solid research foundation had been erected in this domain, encompassing insights from various countries, institutions, and scholars throughout the COVID-19 pandemic. The primary objective of these studies was to investigate the correlation between resilience and nurses’ mental health, PTSD, and job satisfaction, along with corresponding intervention strategies. Considering the persisting pressures and challenges confronting nurses, further research is paramount to effectively ameliorate their overall health and health, based on this newly established research framework.

## 6. Limitations

The current investigation exclusively concentrated on the evaluation of nurses’ psychological resilience within the Web of Science database, excluding research data from other sources. This exclusivity was necessitated by the current constraint of Citespace software, which solely permits the importation of data from a single database. However, it was imperative to highlight that Citespace software exhibits superior efficiency in analyzing data derived from the Web of Science,^[59,60]^ thereby validating our decision to utilize data exclusively from this particular database for our analytical purposes. To address this limitation, future research could explore integrating data from multiple databases to obtain a more comprehensive and diverse research perspective. At the same time, researchers can also consider using other analytical tools or methods to process data from different databases to ensure the comprehensiveness and reliability of research results.

## Author contributions

**Conceptualization:** Neng Liu.

**Data curation:** Neng Liu.

**Formal analysis:** Jindong Yi.

**Funding acquisition:** Fulai Yuan.

**Methodology:** Jindong Yi.

**Resources:** Jindong Yi.

**Supervision:** Fulai Yuan.

**Validation:** Pan Su.

**Visualization:** Pan Su.

**Writing – original draft:** Pan Su.

**Writing – review & editing:** Pan Su.
